# Associations between occupational stress, burnout and well-being among manufacturing workers: mediating roles of psychological capital and self-esteem

**DOI:** 10.1186/s12888-017-1533-6

**Published:** 2017-11-15

**Authors:** Ziyue Wang, Hongbo Liu, Haijian Yu, Yanwen Wu, Shuai Chang, Lie Wang

**Affiliations:** 1 Department of Social Medicine, School of Public Health, China Medical University, No.77 Puhe Road, Shenyang North NewArea, Shenyang, Liaoning People’s Republic of China; 2 School of Public Health, China Medical University, No.77 Puhe Road, Shenyang North New District, Shenyang, Liaoning People’s Republic of China

**Keywords:** Occupational stress, Burnout, Well-being, Positive psychology

## Abstract

**Background:**

Occupational stress is an important risk factor for mental health among occupational population. Exploring related mediators of workers’ mental health are important to improve their health and performance. Our study aims to explore the relationships between work stress, positive psychological resources, burnout and well-being.

**Methods:**

The study was performed during the period of June and July in 2015. A questionnaire that consisted of the Effort-reward Imbalance Scale, the Maslach Burnout Inventory-General Survey, the Psychological Capital Questionnaire, the Rosenberg Self-esteem Scale, the Flourishing Scale, as well as demographic and working factors.

**Results:**

Psychological capital mediated the associations of effort-reward imbalance and emotional exhaustion, cynicism, professional efficacy and well-being. Self-esteem mediated the associations of effort-reward imbalance ratio with cynicism, professional efficacy and well-being, and mediated the associations of overcommitment and cynicism, professional efficacy and well-being.

**Conclusions:**

The findings indicated that enhancing positive psychological resources could be considered in developing intervention strategies for psychological health among manufacturing employees.

**Electronic supplementary material:**

The online version of this article (10.1186/s12888-017-1533-6) contains supplementary material, which is available to authorized users.

## Background

A growing number of companies start to pay attention to not only the physical health of their employees, but also their psychological health. Occupational stress exists in all occupations. However, excessive occupational stress has been considered “toxic” parts of job environment, and is often associated with psychological and physical health. Empirical studies demonstrated that occupational stress was a risk factor for burnout, poor wellbeing, cardiovascular disorders [1–3]. Research on the relationships between occupational stress and mental health has predominantly focused on medical staff, teachers, and information technology employees [4, 5]. Meanwhile, prior studies of manufacturing workers have mostly concentrated on the health hazards of occupational exposure [6] and neglected the research of their psychological health. To fill this gap, this study aimed to explore the level of occupational stress in manufacturing workers and its potential mechanism whereby their psychological health is affected.

The well-known model of effort-reward imbalance (ERI), proposed by Siegrist, was adopted to understand the potential mechanism of stress [7, 8]. This model consisted of three core parts: efforts, rewards and overcommitment, and emphasized the lack of reciprocal social exchange between costs (efforts, such as time pressure to fulfill a task) and gains (rewards, such as money, respect/support received from colleagues, job security, and career opportunities) at work. The imbalance between effort and reward often lead to emotional distress. The model also introduced a specific pattern of coping with job demands, termed as overcommitment, referring to “a set of attitudes, behaviors and emotions reflecting excessive striving in association with a strong desire of being approved and esteem”. The overcommitted individuals may make great efforts, even under low-gain circumstances, and thus were more likely to experience burnout [9].

Burnout is regarded a syndrome that can have significant effects on physical and psychosocial well-being [10–12]. The popular theory of job burnout was developed by Maslach, Schaufeli and Leiter [13], who defined burnout as “a psychological syndrome in response to chronic interpersonal stressors on the job” or a set of negative consequences of prolonged work stress [12]. The three key dimensions of this syndrome are an overwhelming exhaustion, a sense of cynicism and detachment from the work, and feelings of ineffectiveness or lack of accomplishment [13]. The general survey of Marlsh Burnout Inventory (MBI-GS), which is based on the three-dimensional of burnout, has been largely shapes and dominates burnout research [14]. On the level of organization, burnout is associated with turnover intention, lower productivity, and a decreased commitment [11, 15]. Wang et al. have been demonstrated that teachers who experienced high level of occupational stress would have more symptoms of burnout [16]. Job burnout is a psychological syndrome arising in response to chronic interpersonal stressors and job tediousness [11]. Similarly, depression is one of the psychological stresses that may overlap with burnout. However, the distinction between burnout and depression is that burnout is supposedly work related, whereas depression is considered to be more multifactorial in origin and pervasive in nature [17]. Futhermore, Shin et al. [12] proposed that burnout was one of the strongest predictors of depression by a longitudinal study. Therefore, this study used burnout to understand the negative mental status of occupational population. In addition, the World Health Organization [18] argued that the focal point of mental well-being should not be limited to the absence of mental illness, but the positive character of mental system and the attitudes that are inherent to it. Employees with high efforts and low rewards had higher risks of poor well-being [19]. Eudaimonic well-being, one of the main theoretical frameworks that has been proposed, refers to “the dimensions that describe the optimal psychological functioning of the individuals” [20]. Diener et al. [21, 22] developed the Flourishing Scale (FS) to provide a comprehensive and brief measure of flourishing that summarized the current dimensions proposed by others authors, and an overview of the individual’s perception of person’s own positive functioning. The items of the FS describe broad and important facets of human functioning: engagement and interest, self-acceptance, competence, optimism, meaning and purpose, supportive and rewarding relationships, perception of the personal contribution to the well-being of others, and being respect. The current study used burnout and psychological well-being to explore the associations between occupational stress and psychological health in terms of both negative and positive outcomes. Thus, we hypothesize:

- H1 There is a positive association between job stress and job burnout among workers.
- H2 There is a negative association between job stress and well-being among workers.

Although the associations between ERI and psychological outcomes have been examined in certain occupations, the mechanisms behind the associations still remain largely unknown. Over the years, more and more researchers began to notice that stress reaction differs a lot among different individuals, no matter in mental or physical health. Stress and coping theory considered that personality characteristics could influence individual’s appraisal and coping processes, and even psychological symptoms in stressful situations [23]. Based on the transaction theoretical framework, studies on the process of dealing with stress should consist of not only independent and outcome variables, but also mediating variables that determine the individual more or less successful adaption to the stress [24]. With an increasing recognition of the value of positive psychology, organizations may improve their employees’ physical and mental health by enhancing their inner positive psychological resources. This is consistence with the transactional theory, by which stress is the condition that results when person-environment transactions lead the individual to perceive a discrepancy between the demands of situation and the resources of the person’s psychological, biological or social systems [25]. Psychological capital (PsyCap) is a high-order construct extracted from positive organizational behavior, and has been demonstrated as a positive resource for improving employees’ performance, job satisfaction, well-being, and for decreasing stress, turnover, and burnout [26–29]. PsyCap refers to a positive state of mind exhibited during the growth and development of an individual and consists of four state-like psychological resources: hope, self-efficacy, optimism and resilience, all of which are measurable, developable and manageable [27]. In addition to some studies focused on the antecedents and outcomes of PsyCap, there have been other studies pay attentions on the underlying mechanisms through which PsyCap impacts work stress at different level of analysis. Park et al. [30] found the mediating role played by PsyCap in the association between empowering leadership and psychological well-being. Wang et al. [31] looked at PsyCap as a mediator in linking work-family conflict to job burnout. Drawing on prior researches, this study suggests that PsyCap may mediate the associations of occupational stress with burnout and psychological well-being. Besides PsyCap, self-esteem is also recognized as an important internal resource. It is the evaluation which the individual makes and customarily maintains with regard to him or herself [32]. Rosse et al. [33] considered that low self-esteem individuals may be less effective in interpersonal relationships, and experience feeling of incompetence in their relationships with colleagues. Low self-esteem individuals also may have fewer psychic resources to cope with daily stressors, making them vulnerable to emotional exhaustion. Previous studies have found that self-esteem is an important factor in alleviating symptoms of burnout [34]. Self-esteem is an important inner resource in dealing with stress, and is also one of several mediating variables that influence the effects of stressors on health consequences. Based on the above, we formulate the following hypotheses:

- H3: PsyCap mediates association between job stress and burnout.
- H4: PsyCap mediates association between job stress and well-being.
- H5: Self-esteem mediates association between job stress and burnout.
- H6: Self-esteem mediates association between job stress and well-being.

**Fig. 1:**
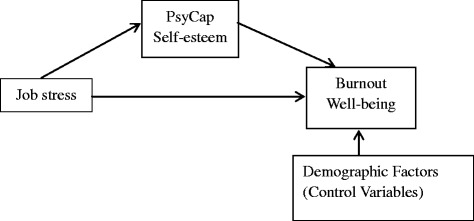
Conceptual framework of this study. PsyCap:psychological capital

As described above, the present study aims to investigate the associations between occupational stress, PsyCap, self-esteem, burnout and well-being, as well as the potential mediating roles of positive psychological resources.Differ from earlier studies about work-related stress that forcus on the single intervening variable, the present study adopt multi- intermediary test to build a systematic theoretical framework of stress and stress reaction from the perspective of positive psychology. It is helpful for management decision-making on manufacturing workers, enriching the existing theoretical model of positive psychology, and acting as preventive strategies during working lifetime (Fig. 1).

## Methods

### Study design and sample

A cross-sectional study was conducted in Northern region of China in June and July, 2015. In total, 26 factories (1500 workers) were randomly selected by cluster sampling in an industry which made component parts of airplanes. Trainees were excluded in this study. Self-administered questionnaires were directly distributed to these subjects after obtaining their written informed consents. Each worker was given a questionnaire to read declaration of private information secrecy and complete voluntarily. Investigators were responsible for distributing, retrieving and managing the questionnaires, and they provided explanation without inducement for any unclear questionnaire items. The average questionnaire spent 10 to 15 min. 79 workers refused to participate in the study because of reading difficulty. The questionnaires with missing data that accounted for more than 15% of the whole scale were regarded invalid. Finally, effective responses were obtained from 1219 individuals (effective response rate: 81.3%), with 923 (75.7%) males and 296 (24.3%) females. The study was approved by the Committee on Human Experimentation of China Medical University (Additional file 1).

### Demographic and working characteristics

Demographic information regarding age, gender, marital status, and education level were obtained. Three working characteristics were also measured: Monthly income, years of experience, and weekly working hours.

### Measurement of occupational stress

Occupational stress was measured by the Chinese version of the effort-reward imbalance (ERI) model [35]. The ERI questionnaire consists of three dimensions: extrinsic effort (6 items), reward (11 items), and overcommitment (6 items). The items of extrinsic effort and reward are scored on a 5-point scale, and higher scores indicated higher demands of efforts and higher rewards. For the overcommitment subscale, responses are scored from 1 to 4, with higher scores indicating higher demands characterized by excessive work-related commitment. Effort-reward ratio (ERR) was calculated using a predefined algorithm that quantifies the degree of mismatch between high cost and low gain, with a correction factor of 0.5454 [8]. The Cronbach’s coefficients for extrinsic effort, reward, and overcommitment scales were 0.911, 0.936, and 0.771 in the present study.

### Measurement of psychological capital

PsyCap was evaluated by the Chinese version of the 24-item Psychological Capital Questionnaire (PCQ) [27]. The PCQ is comprised of four dimensions: self-efficacy (6 items), hope (6 items), resilience (6 items), and optimism (6 items). Each question is scored from 1 (strongly disagree) to 6 (strongly agree). Higher scores indicate higher levels of psychological capital. The PCQ has demonstrated adequate reliability and validity in multiple samples [27, 36]. For the total scale in our study, the Cronbach’s alpha coefficient was 0.953.

### Measurement of self-esteem

Self-esteem was measured with Rosenberg self-esteem scale (RSES), developed by the sociologist Dr. Morris Rosenberg [32]. This scale is a ten-item questionnaire. Each item is scored on a 4-point Likert scale, which ranged from “strongly agree” to “strongly disagree”. Five of the items are positively stated and five are negatively stated. The Chinese version was translated by Cheng et al. [37] and has been widely used in different populations. In the current study, The Cronbach’s alpha coefficient was 0.879.

### Measurement of burnout

This study used Maslach Burnout Inventory-General Survey (MBI-GS) to measure burnout [38, 39]. The scale consists of three dimensions: emotional exhaustion (EE), cynicism (CY), and professional efficacy (PE). Each of the items is scored on a Likert scale from 0 to 6. Higher scores on the EE and CY, and lower score of PE indicate higher level of burnout. The Chinese version of the MBI-GS was revised and validated by Li et al. and has demonstrated good reliability and validity in the Chinese population [40]. In this study, the Cronbach’s alpha coefficients for EE, CY, and PE were 0.940, 0.940, and 0.928.

### Measurement of well-being

Psychological well-being was measured with the brief 8-item Flourishing Scale (FS). The range of scores is from 8 to 56, where higher scores mean a higher level of psychological well-being [41]. The FS scale has demonstrated good validation in different cultures [42]. The Cronbach’s alpha coefficient was 0.950 in this study.

### Statistical analyses

Frequency statistics and one-way ANOVA or t-test was used to describe distributions of burnout and well-being in categorical demographic and working characteristics with mean, standard deviation (SD), number (N) and percentage (%). Pearson’s correlation was used to examine correlations among major variables. Hierarchical regression analysis was adopted to explore the effects of independent variables on burnout and well-being. In step1, all demographic and working variables which were significantly related to dependent variables were entered. Dummy variables were set for the categorical variables; in step 2, the ERR and overcommitment were entered; in step 3, positive resources variables were added. Standardized estimate (β), F, R^2^ and R^2^-changes (ΔR^2^) for each step were displayed. Four different models were estimated in this study, one for each dimension of burnout and one for well-being. Asymptotic and resampling strategies, developed by Preacher and Hayes [43], were used to examine the mediating roles of positive psychological resources (a*b product) on the association between occupational stress and psychological outcomes (burnout and well-being). In these regression equations, ERI and overcommitment were modeled as predictors, burnout and well-being as dependent variables, PsyCap and self-esteem as mediators, and demographic and working variables as covariates. The bootstrap estimate was based on 5000 bootstrap samples. The bias-corrected and accelerated 95% confidence interval (BCa 95% CI) for each a*b product was calculated, and a BCa 95% CI excluding 0 indicated a significant mediating role. All analyses were performed using SPSS 17.0 for Windows, and the significance level was set at *p* < 0.05(two-tailed). Missing values were addressed with mean substitution.

## Results

### Descriptive statistics

Demographic and working characteristics of the workers and univariate analysis of burnout and well-being scores were shown in Table 1. The mean age of the subjects was 35.44 years (SD = 9.47, ranged from 20 to 59). Univiariate analyses indicated that age, monthly income, weekly working hours, and working years were associated with emotional exhaustion and cynicism, respectively (*P* < 0.05). Education level was related to cynicism (*P* < 0.05). Age, educational level, and monthly income were related to professional efficacy (*P* < 0.05). Gender, education, income, and weekly working hours were related to well-being (*P* < 0.05).

**Table 1:** Distribution and univariate analyses of MBI-GS and well-being

	N(%)	EE	CY	PE	WB
		Mean ± SD	Mean ± SD	Mean ± SD	Mean ± SD
Age					
20–29	437(35.8)	10.46 ± 7.09	7.08 ± 5.99	26.73 ± 11.18	44.90 ± 8.51
30–39	420(34.5)	10.21 ± 7.76	6.84 ± 6.40	28.56 ± 11.27	44.42 ± 8.76
> 40	362(29.7)	8.41 ± 6.89	5.41 ± 5.34	26.51 ± 12.55	44.61 ± 8.43
*F*		9.042**	8.841**	3.827*	0.338
Gender					
male	923(75.7)	9.82 ± 7.21	6.68 ± 5.94	27.11 ± 11.52	44.36 ± 8.82
female	296(24.3)	9.58 ± 7.65	5.93 ± 6.10	27.87 ± 12.09	45.53 ± 7.68
*T*		0.486	1.894	−0.969	−2.181*
Marriage					
Married/cohabitation	886(72.7)	9.57 ± 7.35	6.33 ± 5.97	27.39 ± 11.83	44.57 ± 8.61
Single/dicorced/widow	333(27.3)	10.28 ± 7.23	6.96 ± 6.02	27.04 ± 11.21	44.85 ± 8.48
*T*		1.510	1.644	−0.467	0.517
Educational level					
Technical secondary school and the following	232(19.0)	9.37 ± 7.94	6.36 ± 6.22	24.96 ± 12.65	44.06 ± 9.29
Junior college	492(40.4)	10.13 ± 7.62	7.08 ± 6.44	26.91 ± 12.00	44.10 ± 8.63
Bachelor degree or above	495(40.6)	9.58 ± 6.69	5.99 ± 5.34	28.78 ± 10.59	45.47 ± 8.09
*F*		1.109	4.134*	9.033**	3.808*
Income(RMB, yuan)					
< 4000	919(75.4)	10.14 ± 7.44	6.82 ± 6.13	26.85 ± 11.68	44.12 ± 8.90
≧4000	300(24.6)	8.60 ± 6.81	5.53 ± 5.42	28.67 ± 11.51	46.26 ± 7.24
*T*		3.340**	3.450**	−2.356*	−4.179**
Weekly working hours					
< 40	840(68.9)	8.84 ± 6.86	6.07 ± 5.67	27.30 ± 11.83	45.08 ± 8.47
≧40	379(31.1)	11.81 ± 7.89	7.45 ± 6.55	27.28 ± 11.29	43.70 ± 8.73
*T*		−6.346**	−3.557**	0.031	2.609*
Working years					
< 12	674(47.1)	10.50 ± 7.21	6.97 ± 5.95	27.26 ± 11.32	44.94 ± 8.62
≧12	645(52.9)	9.11 ± 7.36	6.09 ± 6.00	27.33 ± 11.96	44.38 ± 8.52
*T*		3.323**	2.570**	−0.113	1.137

*EE* emotional exhaustion, *CY* cynicism, *PE* professional efficacy, *WB* well-being

*p < 0.05, **p < 0.01 (two-tailed)

### Correlations among continuous variables

The correlations among ERI, PsyCap, self-esteem, components of burnout, and well-being were shown in Table 2. As revealed in the table, ERR, overcommitment were positively correlated with emotional exhaustion and cynicism, respectively, and had negative correlations with professional efficacy, well-being and positive psychological variables. Psycap and self-esteem were negatively related to emotional exhaustion, cynicism and positively related to professional efficacy and well-being.

**Table 2:** Correlations among dimension of MBI-GS, ERI and positive psychological variables

variables	Mean	SD	1	2	3	4	5	6	7	8
1. Emotional exhaustion	9.76	7.32	1							
2. Cynicism	6.50	5.99	.751**	1						
3. Professional efficacy	27.29	11.65	−.140**	−.271**	1					
4. Well-being			−.322**	−.399**	.306**	1				
5. ERR	0.76	0.58	.612**	.614**	−.193**	−.251**	1			
6. Overcommitment	13.42	3.45	.502**	.371**	−.070*	−.257**	.481**	1		
7. PsyCap	4.31	0.80	−.390**	−.486**	.405**	.616**	−.350**	−.279**	1	
8. Self-esteem	21.39	4.65	−.262**	−.357**	.419**	.522**	−.248**	−.285**	.630**	1

*ERR* effort-reward ratio, *PsyCap* psychological capital

*p < 0.05, **p < 0.01 (two-tailed)

### Associations among ERI, PsyCap, self-esteem, and burnout

All independent variables were correlated with the three components of burnout in univariate analyses. Thus, they were entered in the multiple hierarchical linear regression models. Results were presented in Table 3. In step3, after adjusting for age, gender, education, income, working years, and weekly working hours, ERR, overcommitment, and PsyCap were associated with emotional exhaustion. All variables could explain 47.7% of the variance in emotional exhaustion, and positive psychological variables were responsible for an additional 2.6% in Step3. ERR and PsyCap and self-esteem were associated with cynicism and were responsible for 47.3% of the variance in cynicism. ERR, overcommitment, PsyCap, self-esteem were associated with professional efficacy, and could explain 22.8% of its variance.

**Table 3:** Hierarchical linear regression analyses of the factors associated with MBI-GS scores

variables	EE			CY			PE			WE		
	Step1	Step2	Step3	Step1	Step2	Step3	Step1	Step2	Step3	Step1	Step2	Step3
Age	0.041	0.015	0.008	0.002	−0.014	−0.036	0.038	0.04	0.088	−0.115	−0.099	−0.047
Gender	0.003	0.018	0.016	−0.042	−0.03	−0.026	0.01	0.007	−0.011	0.048	0.04	0.026
Education_1	−0.012	−0.022	−0.023	0.013	−0.001	0.002	0.082*	0.088*	0.075*	0.003	0.005	−0.005
Education_2	−0.081	−0.047	−0.034	−0.094*	−0.056	−0.023	0.152**	0.138**	0.075*	0.075	0.062	−0.013
Income	−0.063	−0.061**	−0.041	−0.066*	−0.061**	−0.024	0.06*	0.058*	0.003	0.101**	0.100**	0.024
working hours	0.264**	0.089**	0.096**	0.159**	0.001	0.012	−0.015	0.031	0.017	−0.081**	0.004	−0.017
Working years	−0.189**	−0.105	−0.106*	−0.131	−0.052	−0.048	−0.043	−0.066	−0.082	0.114	0.075	0.063
ERR		0.456**	0.407**		0.553**	0.47**		−0.211**	−0.109**		−0.159**	0.001
Overcommitment		0.258**	0.235**		0.103**	0.048		0.02	0.117**		−0.18**	−0.062*
PsyCap			−0.193**			−0.268**			0.219**			0.466**
Self-esteem			0.033			−0.055*			0.286**			0.208**
F	19.012**	110.79**	99.953**	10.297**	88.214**	98.594**	3.305**	8.113**	32.336**	4.930**	15.806**	78.308**
R^2^	0.099	0.451	0.477	0.056	0.396	0.473	0.019	0.057	0.228	0.028	0.105	0.416
R^2^-change	0.099	0.352	0.026	0.056	0.340	0.077	0.019	0.038	0.171	0.028	0.078	0.311

Education_1 means “Junior college” vs. “Technical secondary school and the following”, Education_2 means “Bachelor degree or above” vs. “Technical secondary school and the following”; *P < 0.05; **P < 0.01(two-tailed)

### Associations between ERI, PsyCap, self-esteem, and well-being

After adjusting for controlling variables which were related to well-being in univariate analyses, as shown in Table 3, ERR and overcommitment were associated with well-being in step 2. In step 3, PsyCap and self-esteem were positively and significantly associated with well-being, respectively. In addition, the effect of ERR and overcommitment on well-being reduced in step3.

### Mediating effects of PsyCap and self-esteem

The indirect pahways of ERI with psychological outcomes through PsyCap and self-esteem are shown in Table 4. The path coefficients of ERI with positive psychological variables (a), the relations of positive psychological variables with dependents (b), the direct relations of ERI with dependents(c), the relations of ERI with dependents after controlling for the effects of the positive psychological variables (c’), a*b (the indirect effects of ERI on the dependent variables through the positive psychological variables) products, and BCa 95% CI for these products are shown in Table 4. BCa 95% CI excluding 0 indicated a significant mediating role. If the indirect path coefficient (c’) was significant (*P* < 0.05), the positive psychological variable would play a part of the intermediary role between independent and dependent variables. If not (*P* > 0.05), it would have wholly mediator effect between independent and dependent variables.

**Table 4:** Intermediary function test

X	M	Y	c	a	b	c’	a*b(BCa95%)
Model1							
ERR	PsyCap	EE	5.8505**	−0.3879**	−1.7822**	5.2241**	0.6914(0.4137,1.0648)
	Self-esteem			−1.1191**	0.0580		−0.0649(−0.2154,0.0337)
Overcommitment	PsyCap	EE	0.5641**	−0.0372**	−1.7822**	0.5148**	0.0662(0.0353, 0.1087)
	Self-esteem			−0.2912**	0.0580		−0.0169(−0.0477,0.0106)
Model2							
ERR	PsyCap	CY	5.7204**	−0.3879**	−1.9967**	4.8659**	0.7746(0.4721,1.1169)
	Self-esteem			−1.1191**	−0.0714*		0.0799(0.0023,0.1980)
Overcommitment	PsyCap	CY	0.1839**	−0.0372**	−1.9967**	0.0889*	0.0742(0.0406, 0.1166)
	Self-esteem			−0.2912**	−0.0714*		0.0208(0.0003,0.0459)
Model3							
ERR	PsyCap	PE	−4.2256**	−0.3879**	3.1443**	−2.1995**	−1.2198(−1.9028,-0.6473)
	Self-esteem			−1.1191**	0.7205**		−0.8063(−1.3403,-0.3646)
Overcommitment	PsyCap	PE	0.0661	−0.0372**	3.1443**	0.3927**	−0.1168(−0.1913,-0.0620)
	Self-esteem			−0.2912*	0.7205**		−0.2098(−0.3089,-0.1346)
Model4							
ERR	PsyCap	Well-being	−2.3444**	−0.3879**	4.9939**	0.0171	−1.9374(−2.6831,-1.2118)
	Self-esteem			−1.1191**	0.3789**		−0.4241(−0.7464,-0.1908)
Overcommitment	PsyCap	Well-being	−0.4467**	−0.0372**	4.9934**	−0.1508*	−0.1855(−0.2783,-0.1058)
	Self-esteem			−0.2912**	0.3789**		−0.1103(−0.1675,-0.0622)

*P < 0.05, **P < 0.01

a: associations of ERI with PsyCap and self-esteem

b: associations of PsyCap/ self-esteem with burnout/well-being

c: direct associations of ERI with burnout/well-being

c’: indirect associations of ERI with burnout/well-being

age, gender, income, working hours, working years and education level are covariates

Given that, for Model 1 (Table 4), PsyCap were mediated the association of ERR with emotional exhaustion (a*b = 0.6914, BCa 95% CI: 0.4137, 1.0648), and mediated the association of overcommitment with emotional exhaustion (a*b = 0.0662, BCa95%CI: 0.0353, 0.1087). PsyCap played a partial mediating effect in the relationship between ERR/overcommitment and emotional exhaustion (c’ = 5.2241, *P* < 0.01). Similarly, for Model 2, PsyCap and self-esteem partially mediated the relationship between ERR and cynicism, and partially mediated the relationships between overcommiment and cynicism. For Model 3, PsyCap and self-esteem were partially mediated the relationship between ERR and professional efficacy, and partially mediated the relationship between overcommitment and professional efficacy.

In the Model 4, there were significant and full mediating roles of PsyCap and self-esteem between ERR and well-being (both of the mediators’ Bca95%CI excluded 0, c’ = 0.0171, *P* > 0.05). PsyCap and self-esteem partially mediated the associations between overcommitment and well-being (both of the mediators’ Bca95%CI excluded 0, c’ = −0.1508, *P* < 0.05).

## Discussion

This study enriched the theoretical framework of positive psychology in terms of the associations between occupational stress, positive psychological resources and mental health outcomes, as well as the potential mediating roles of positive psychological resources in the relationships. According to the results of multiple hierarchical regressions, lower levels of education and income, longer weekly working hours, and shorter working years were associated with burnout. Older workers were less likely to have symptoms of emotional exhaustion, which was in line with the findings of previous studies [4]. They had more experience than younger workers and were more likely to better deal with different situations. Workers with less working years often begin with great expectations and may go through a difficult adjustment period, due to the lack of experience and practical skills. Above all, the three variables of ERI, PsyCap and self-esteem explained a large proportion of the variance in burnout. For the well-being model, all the demographics indicators had no significant association with well-being when they were controlled. The results suggested that occupational stress and positive psychological resources could have relatively major impacts on employees’ well-being.

The percentage of our sample workers who had occupational stress was 22.4%, which was similar to that reported by university teachers [44], and lower than some other groups of employees [45]. With regard to the relationship between occupational stress and mental health, workers who experienced high ERR and overcommitment had higher level of emotional exhaustion and cynicism, and lower professional efficacy and well-being among the manufacturing workers. These results were consistent with the findings of previous studies [3, 46]. Workers with high effort, such as time and energy, might tend to experience more negative emotions. When the level of extrinsic effort exceeded intrinsic reward, the impact on both physical and mental health could easily lead to burnout and decrease the level of psychological well-being. Based on the Allostatic Load model, Girardi et al. [47] demonstrated that work-related stress could associate with biomarkers of inflammation, through negative affectivity which affected the exposure to psychological stressors. In addition, most of the manufacturing workers are often exposed to adverse working environment such as noise, high temperature, dust, organic solvents, and prolonged working hours on the assembly line with monotonous and repetitive tasks. These adverse factors may impact the mental health, and contribute to their burnout [48]. There is no doubt that the objective working conditions are unlikely to change within a foreseeable future. Therefore, measures and strategies improving the inner psychological resources of the manufacturing workers should be introduced in psychological health interventions.

An increasing number of scholars have become interested in the associations between positive psychological resources and mental health outcomes. PsyCap can help employees fight against stressors and work more effectively. It has been reported that individuals with higher levels of PsyCap are able to have more confidence and make greater efforts to pursue success, preserve the will to accomplish tasks or goals, bounce back from adversity or personal setbacks, and perceive positive expectations and attributes regarding consequences [27, 49]. In the present study, PsyCap was found to be negatively associated with emotional exhaustion and cynicism, respectively, and positively associated with professional efficacy and well-being among the manufacturing workers. As another important inner resource for psychological and social functioning, self-esteem was found to be negatively correlated with cynicism, and positively correlated with professional efficacy and well-being, which were consistent with the findings in previous studies [50, 51]. Low self-esteem has been found to be a risk or vulnerability factor for mental health. These results contributed to the understanding that PsyCap and self-esteem were positive resources for combating burnout and encouraged us to explore the mediating roles of positive psychological resources in the relationship between occupational stress and burnout in this population.

Results revealed that PsyCap played a mediating role in the relationships between ERI and emotional exhaustion and cynicism, and also partially mediated the effects of ERR on professional efficacy. Self-esteem partially mediated the effects of ERI on cynicism and partially mediated the effects of ERR on professional efficacy. Both PsyCap and self-esteem mediated the relations between ERI and well-being. Workers who perceived more effort-reward imbalance and overcommitment would be more likely to experience lower levels of positive psychological resources which in turn increased the possibilities of developing burnout symptoms and decreasing their well-being. Compared with decreasing workers’ occupational stress, it is a more positive and strategic way for companies to develop programs to increase the inner positive resources of their workers, thus to enhance the mental health and improve the organizational performance in the long run.

The findings have theoretical and practical implications for occupational health and performance management. In theory, different from previous studies which focused on the effects of single intervening organizational behavior variable, our study explored the concurrent effects of positive psychological resources on both negative and positive mental health constructs, and revealed that PsyCap and self-esteem might be positive resources for combating stress symptoms. In the competitive society, mental health issues of employees are becoming more common, and psychological health is a basic requirement for the staff to bring their intelligence into play. Organizational managers usually care more about advanced technologies and skills than positive inner resources of their staff to improve organizational performance. According to the theory of the positive psychology, positive psychological capital is open to be developed and managed, including optimism, self-efficacy, hope, resilience, self-esteem, and some other positive psychological resources. Interventions designed to enhance positive psychological resources have been introduced in previous studies and attracting more and more attention. Therefore, practical strategies of enhancing workers’ positive psychological resources should be developed to help workers better adjust to their working situations.

Several limitations have to be mentioned in this study. The primary limitation of our study is the cross-sectional nature of the data, which do not permit causal conclusions to be drawn. Although positive psychological resources can account for a large part of the predictive associations between occupational stress and mental health, our study cannot confirm a meditational model through cross-sectional design. Longitudinal studies are required to test these hypotheses, along with more complex statistical models. Besides, as a study based on self-report measure, the results cannot rule out other possible factors. Third, we couldn’t neglect that some associations such as the correlations between overcommitment and professional efficacy are statistically significant, but very small. Finally, generalizations from our data should be taken with caution since the sample comprised only a small proportion of all workers in manufacturing industries.

## Conclusions

To summarize, our findings revealed that both extrinsic stress (ERR) and intrinsic stress (overcommitment) were positively associated with emotional exhaustion and cynicism, respectively, among manufacturing workers. Only ERR was negatively associated with professional efficacy. PsyCap mediated the effects of ERI on emotional exhaustion and cynicism, and also mediated the effects of ERR on professional efficacy. Self-esteem mediated the effects of ERI on cynicism, and the effects of ERR on professional efficacy. Both PsyCap and self-esteem mediated the relations between ERI and well-being. Hence, interventions to decrease Chinese manufacturing workers’ occupational stress and to enhance their positive psychological resources should be developed and put into practice.

## Additional file

Additional file 1: Survey questionnaires. (DOCX 11 kb)

## Abbreviations

CY: Cynicism
EE: Emotional exhaution
ERI: Effort-Reward Imbalance
ERR: Effort-Reward Imbalance Rate
FS: Flourishing scale
MBI-GS: Maslach burnout inventory-general survey
OC: Over-Commitment
PE: Professional efficacy
PsyCap: Psychological capital
WB: Well-being
