# Supplementary material for: Associations between occupational stress, burnout and well-being among manufacturing workers: mediating roles of psychological capital and self-esteem
Source: BMC Psychiatry. 2017 Nov 15;17:364. doi: 10.1186/s12888-017-1533-6 (PMC5688661; doi:10.1186/s12888-017-1533-6)
Supplement: Additional file 1: — Survey questionnaires. (DOCX 11 kb) [file 12888_2017_1533_MOESM1_ESM.docx]

Additional file 1

**MBI-GS:** [**http://www.mindgarden.com/117-maslach-burnout-inventory**](http://www.mindgarden.com/117-maslach-burnout-inventory)

ERI scale: <https://www.sciencedirect.com/science/article/pii/S0277953603003514>

PsyCap scale: Luthans F, Avolio BJ, Avey JB, Norman SM. Positive psychological capital: Measurement and relationship with performance and satisfaction. Pers Psychol. 2007;60: 541–572.

Rosenberg self-esteem scale (RSES): <http://www.yorku.ca/rokada/psyctest/rosenbrg.pdf>

Flourishing scale: <http://www.midss.org/sites/default/files/fs.pdf>
